# Giant gauge factor of Van der Waals material based strain sensors

**DOI:** 10.1038/s41467-021-22316-8

**Published:** 2021-04-01

**Authors:** Wenjie Yan, Huei-Ru Fuh, Yanhui Lv, Ke-Qiu Chen, Tsung-Yin Tsai, Yuh-Renn Wu, Tung-Ho Shieh, Kuan-Ming Hung, Juncheng Li, Duan Zhang, Cormac Ó Coileáin, Sunil K. Arora, Zhi Wang, Zhaotan Jiang, Ching-Ray Chang, Han-Chun Wu

**Affiliations:** 1 School of Physics, Beijing Institute of Technology, Beijing, P. R. China; 2 Department of Physics, National Taiwan University, Taipei, Taiwan; 3 Department of Chemical Engineering & Materials Science, Yuan Ze University, Taoyuan City, Taiwan; 4 Department of Applied Physics, School of Physics and Electronics, Hunan University, Changsha, P. R. China; 5 Graduate Institute of Photonics and Optoelectronics and Department of Electrical Engineering, National Taiwan University, Taipei, Taiwan; 6 Department of Intelligent Robotics Engineering, Kun-Shan University, Tainan, Taiwan; 7 Department of Electronics Engineering, National Kaohsiung University of Science and Technology, Kaohsiung, Taiwan; 8 Elementary Educational College, Beijing key Laboratory for Nano-Photonics and Nano-Structure, Capital Normal University, Beijing, P. R. China; 9 Centre for Research on Adaptive Nanostructures and Nanodevices (CRANN) and Advanced Materials and Bioengineering Research (AMBER), School Chemistry, Trinity College Dublin, Dublin, Ireland; 10 Centre for Nanoscience and Nanotechnology, Panjab University, Chandigarh, India

**Keywords:** Electronic properties and materials, Nanosensors, Sensors, Electronics, photonics and device physics

## Abstract

There is an emergent demand for high-flexibility, high-sensitivity and low-power strain gauges capable of sensing small deformations and vibrations in extreme conditions. Enhancing the gauge factor remains one of the greatest challenges for strain sensors. This is typically limited to below 300 and set when the sensor is fabricated. We report a strategy to tune and enhance the gauge factor of strain sensors based on Van der Waals materials by tuning the carrier mobility and concentration through an interplay of piezoelectric and photoelectric effects. For a SnS_2_ sensor we report a gauge factor up to 3933, and the ability to tune it over a large range, from 23 to 3933. Results from SnS_2_, GaSe, GeSe, monolayer WSe_2_, and monolayer MoSe_2_ sensors suggest that this is a universal phenomenon for Van der Waals semiconductors. We also provide proof of concept demonstrations by detecting vibrations caused by sound and capturing body movements.

## Abstract (web summary)

The Gauge factor (GF) enhancement in strain sensors remains a key challenge. Here the authors leverage the piezoelectric and photoelectric effects in a class of van der Waals materials to tune the GF, and obtain a record GF up to 3933 for a SnS_2_-based strain sensor.

## Introduction

Electronic strain gauge sensors were invented by E. E. Simmons and A. C. Ruge in 1938 to measure the strain and deformation experienced by objects. Recently, due to the rapid development of soft robots, remote monitoring, artificial intelligence, and wearable health care devices, there has been surge in demand for high-flexibility, high-sensitivity, and low-power strain gauges that can monitor and sense small deformations and vibrations in extreme conditions^1–16^. The sensitivity metric of a strain gauge, known as the gauge factor (GF), is defined as:

1 $${\mathrm{GF}} = \frac{1}{{R_0}}\frac{{dR}}{{ds}} + \left( {1 + 2v} \right)$$

Where *s* is the applied strain, *R* is the resistivity and *R*_0_ is the material’s zero-strain resistivity, and *ν* is the Poisson ratio^6,12,17^. Most commercially available sensors are based on metal foils. However, as metals have no bandgap they undergo only small changes in resistivity with applied strain, which tends to result in low GF values, in the range of 1–5^4,17–19^. Semiconducting materials have nonzero band gaps (*E*_g_) and the carrier density (*n*_i_) of an intrinsic semiconductor is proportional to $e^{\frac{{ - E_{\mathrm{g}}}}{{2k_{\mathrm{B}}T}}},$ where *T* is the temperature, and *k*_B_ is the Boltzmann constant. Applying strain can lead to a change in the band structure, thereby modifying the carrier density and resulting in higher GF values. For p-type silicon, GF values of up to ~175 have been reported^17^. However, bulk semiconducting materials are often brittle, restricting their use to a narrow sensing ranges, i.e., device failure occurs at low strain^20–25^. On the other hand, many Van der Waals layered materials (VdWLMs) display high elasticity and Young’s moduli, coupled with properties that are highly sensitive to external deformations while being able to withstand very large localized deformations without breaking^26–30^. Moreover, VdWLMs also display piezoresistive properties suggesting they could find use as active components in assorted electromechanical sensors, such as strain gauges, as well as microelectromechanical and nanoelectromechanical systems (MEMS and NEMS)^4,6,17,27,31–34^. For example, MoS_2_, a semiconducting Van der Waals material, has been shown to have two negative gauge factors ranging from −225 for bilayers to −50 for few-monolayer samples^27,35^. As noted, VdWLMs are known to be very sensitive to their environment and fabrication methods^30^. Moreover, the GF of Van der Waals material strain sensors are usually fixed once fabricated^2,3,6,7,26,27,32^. A significant level of effort has been devoted toward tuning and enhancing the GF of Van der Waals materials based strain sensors, such as by doping Van der Waals materials with vanadium^19^, direct control by gating^35^, decreasing layer number^4,27^, and controlling the strain direction^32^. Typically, carrier doping during fabrication or gas absorption will affect the GF of a sensor. In spite of these efforts, the GF values reported for various types of strain sensors are normally limited to well below 300^3,6,7,19,26–28,32,35^ and the range over which they can be tuned using a gate voltage is again quite restricted^35^.

In this work, we report a strategy to tune and enhance the GF of strain sensors based on VdWLMs by controlling the carrier density and mobility via photo excitation. A gauge factor of as high as 3933 is found for a SnS_2_ based strain sensor, which is five times higher than the GF value reported for strain sensors based on VdWLMs and exceeds the GF value reported for ZnSnO_3_ nanowires^4,9^. Moreover, the GF can also be tuned over a wide range of values, between 23 and 3933. Our observations indicate that it is a universal phenomenon for Van der Waals semiconductor materials, demonstrated by measurements on a representative group of materials including SnS_2_, GaSe, GeSe, monolayer WSe_2_, and monolayer MoSe_2_. We also provide two real world demonstrations for our sensors, by using a prototype to detect tiny vibrations caused by sound and the normal daily movement of a human body.

## Results

### Giant gauge factor of SnS_2_ based strain sensors

**Fig. 1:**
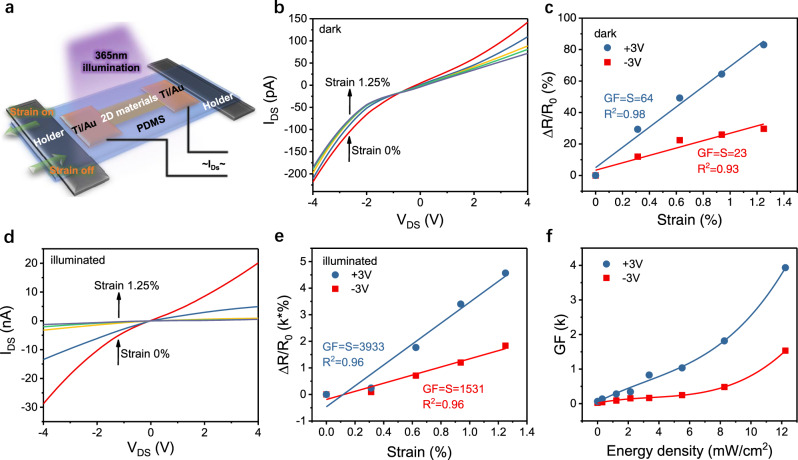
Giant gauge factor (GF) of SnS_2_ based strain sensors. **a** Schematic of the Van der Waals strain sensor devices. **b** *I–V* curves under various strain conditions measured in darkness. **c** The variation of $\Delta R/R_0$ as a function of strain for the sensor measured in darkness. **d** *I–V* curves under various strain conditions measured with 365 nm light illumination. **e** The function of $\Delta R/R_0$ versus strain of the sensor measured under 365 nm illumination. **f** GF value of the strain sensor as a function of the power density of 365 nm illumination.

Figure 1a shows a schematic of a Van der Waals strain sensor device. To prepare the devices, the VdWLMs are transferred onto polydimethylsiloxane (PDMS) substrates. Details of the device fabrication can be found in the Supplementary Information. The characterization of the strain devices was performed utilizing a commercial strain-testing platform in atmosphere at room temperature. The strain was applied by elongating or bending the PDMS substrates. Since the PDMS substrate and Van der Waals material sensor are bonded together with the metal electrodes, the strain experienced by the sensors will be approximately equal to that applied to the PDMS substrate^33,36–38^. In order to inject nonequilibrium carriers, a 365 nm light was used to excite electron-hole pairs in the VdWLMs. Supplementary Fig. 1 shows an optical image of a SnS_2_-based strain sensor. The thickness D of the crystalline SnS_2_ flake is around 60 nm and the distance between electrodes is around 30 µm. Figure 1b plots the characterization of *I–V* behavior of the SnS_2_-based strain sensor under various strain conditions measured in darkness. The *I–V* curves are asymmetric and show distinct nonlinear behavior, indicating a Schottky barrier is formed between SnS_2_ and metal electrodes. Moreover, the *I–V* curves shift downward with applied tensile strain, suggesting strain can modify the electrical transport properties of the sensor. It is worth noting that the Schottky contacted devices are reported to have much greater responsivity than Ohmic contacted devices.^39–41^ To evaluate the device performance, $\Delta R/R_0$ versus strain is plotted and fitted linearly in Fig. 1c, where the slope value is the GF of the strain sensor at a 3 V bias is shown. For a positive bias of 3 V the GF is 64, but a value 23 is observed for a reversed bias voltage (−3 V). To investigate the effect of the photo excitation, Fig. 1d plots the *I–V* behavior of the same SnS_2_-based strain sensor measured over the same strain range under illumination by a 365 nm light. The plots follow a behavior similar to that observed under dark conditions, but much greater variation is exhibited. From a linear fit of the $\Delta R/R_0$ as a function of strain in Fig. 1e we find that the GF value is 3933 for a bias voltage of +3 V, and 1531 for −3 V. To the best of our knowledge, the observed GF value significantly exceeds known GF levels reported for sensors functioning in ambient conditions, in the semiconductor field. For PtSe_2_ nanosheets a GF value of −85 has been achieved^7^, while the GF value for a monolayer MoS_2_ device of ~760 is the highest reported in the field of VdWLMs^4^, a value of 2820 has been achieved for an InAs nanowire^31^ and 3740 for a ZnSnO_3_ microwire^9^. Our value is five times higher than that for monolayer MoS_2_, and even exceeds the value for ZnSnO_3_ microwire sensors. To further illustrate the effect of photo illumination on the GF value of the SnS_2_ strain senor, we illuminated the sensor with different power densities. The obtained GFs are summarized in Fig. 1f. Remarkably, photo illumination can significantly enhance the performance of SnS_2_ based strain sensors. The intensity can tune the GF over a large extended range, from 56 to 3933. An even greater GF value can be expected for illumination with higher power intensities as we did not observe saturation of the behavior for even the highest power density available with our light source. We have measured more than 20 devices and all devices show similar behavior. Moreover, the GF recovers fully when the illumination is turned off. The stability of the strain sensors with laser illumination was also carefully investigated. Supplementary Fig. 1b shows the current response of a SnS_2_ based sensor measured under 365 nm light illumination over many cycles of repeated stretching. The current reached is almost the same value during each stretching cycle and it then recovers when the strain is released.

### Origin of enhanced GF value by photo illumination

The downward shift of the nonlinear *I–V* curves in Fig. 1b and d clearly indicate that the carrier transport through the SnS_2_/metal interfaces and SnS_2_ channel region is affected by the strain as well as by the photo illumination. The device band profile is illustrated in Supplementary Fig. 2 and considering the electrons flowing from the left-hand side to the right-hand side, the reverse-biased diode at the first contact (1) dominates the device current. There are three transport mechanisms for majority carriers to cross a metal-semiconductor contact junction: (i) diffusion of carriers from the semiconductor into the metal, (ii) thermionic emission of carriers across the Schottky barrier, and (iii) quantum-mechanical tunneling through the barrier^42–44^. For a low-doped semiconductor or at high temperature the tunneling current is comparatively negligible^45^. In these situations, the transport of majority carriers in sensor device mainly results from diffusion and thermionic emission, and the current through the device can be described by classical thermionic emission-diffusion theory^8,9^:

2 $$I =	 \, SA^{ \ast \ast }T^2{\mathrm{exp}}\left( { - \frac{{\phi _{\mathrm{B}}}}{{k_{\mathrm{B}}T}}} \right){\mathrm{exp}}\left( {\frac{{\root {4} \of {{q^7N_{\mathrm{D}}\left( {V - IR + V_{{\mathrm{bi}}} - k_{\mathrm{B}}T/q} \right)/8\pi ^2\varepsilon _{\mathrm{S}}^3}}}}{{k_{\mathrm{B}}T}}} \right)\\ 	\,\left( {1 - e^{ - q\frac{{V - IR}}{{k_{\mathrm{B}}T}}}} \right)$$

where *S* is the cross-sectional area of the SnS_2_ nanosheet, A** (253.2 A cm^−2^ K^−2^)^46^ is the Richardson constant, *q* is the free electron charge, *N*_D_ (7.575 × 10^19^  m^−3^) (The carrier density *n*_0_ ≈ *N*_D_ is obtained from unstrain-dark current fitting using the measured mobility of 0.00005 m^−2^ V^−1^ s^−1^. Its corresponding Fermi energy is 4.682 eV using the effective conduction-band density of states *N*_C_ = 3.4579 × 10^25^ m^−3^ at room temperature.) is the donor concentration, *V* is the applied voltage, ${\it{\epsilon }}_{\mathrm{s}}$ ($5.6{\it{\epsilon }}_0$) is the dielectric constant of SnS_2_, *ϕ*_B_ is the Schottky barrier height (SBH), and *V*_bi_ (*V*_bi_ = *E*_F_−*ϕ*_M_) is the build-in potential that is the difference of SnS_2_ Fermi energy *E*_F_ (4.682 eV) and metal work function *ϕ*_M_ (4.3 eV)^47^. The channel resistance *R* follows from Ohm’s law

3 $$R = L/qS\left( {p\mu _p + n\mu _n} \right).$$

*L* is the channel length, *p (n)* and *μ*_*p*_ (*μ*_*n*_) are the hole (electron) density and mobility, respectively, and we simply assume $\mu _p \approx \mu _n/4$. From our analysis, we find that at zero strain in darkness or under 365 nm light illumination, ln(*I*) follows *V*^1/4^ behavior (Supplementary Fig. 3) as the diode bias (*V*−*IR*) is greater than 5*K*_B_*T*/*q* but deviates from *V*^1/4^ at small bias (*V* < 0.6 *V*). This suggests that Eq. (2) is effective in its description of the experimental *I–V* curve.

**Fig. 2:**
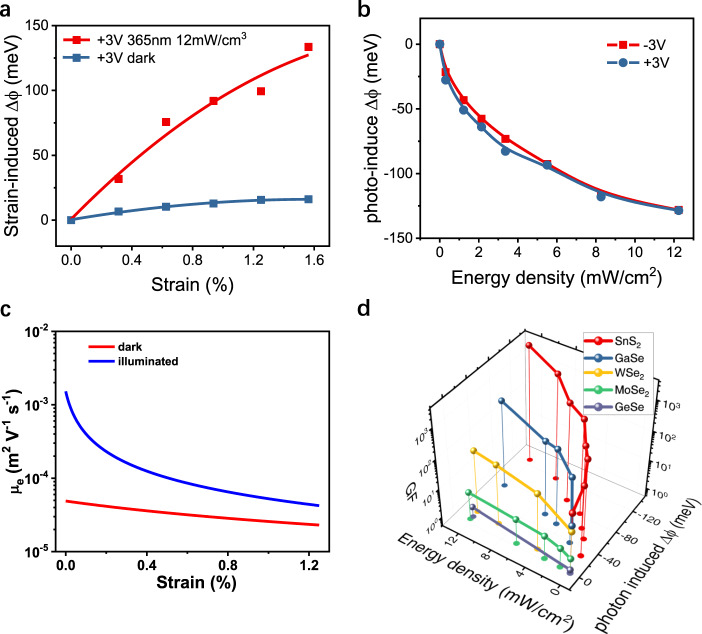
Origin of enhanced GF value by photo illumination. **a** Calculated relative change of equivalent potential variation ($\Delta \phi$) of the SnS_2_ based strain sensor with strain (*s*) with and without external illumination. **b** Calculated relative change of $\Delta \phi$ at zero strain (*s*) as a function of power density of illumination. **c** Calculated mobility as a function of strain in darkness (red line) and under 365 nm laser illumination (blue line). **d** Demonstration of the universality of GF enhancement phenomenon by photo illumination for Van der Waals semiconducting materials including SnS_2_, GaSe, GeSe, monolayer WSe_2_, and monolayer MoSe_2_.

Since S, A**, T, N_D_, and *V*_bi_ are insensitive to strain deformation, the variation of current with strain (*s*) is mainly a result of changes in the SBH and channel resistance (CR) due to the piezoelectric effect. At high bias, the equivalent potential variation (EPV, the exponent part of Eq. (2)) caused by the changes of SBH and CR is given by:

4 $$\Delta \phi = k_{\mathrm{B}}T\ln \frac{{I(0)}}{{I(s)}},$$

where *I*(*s*) is the current at a fixed bias voltage under *s* strain. Figure 2a plots the relative change in $\Delta \phi$ against strain in the high bias voltage range. Note, to show the effect of the strain more clearly, the EPV values in darkness and under illumination have been plotted with respect to their own zero strain condition. We find that EPV increases with strain, more significantly, under laser illumination it increases at a much faster rate than it does in darkness. To further illustrate the effect of laser illumination, the shift of EPV under 365 nm light illumination at zero strain in relation to that in darkness at zero strain can be estimated using:

5 $$\Delta \phi = k_{\mathrm{B}}T\ln \frac{{I_{{\mathrm{dark}}}}}{{I_{{\mathrm{light}}}}}.$$

Figure 2b shows that the EPV at zero strain decreases with increasing the laser power density. In addition, the current responses of a SnS_2_ based strain sensor in a continuous extruding and stretching test, as shown in Supplementary Fig. 4, indicates that the resistance of the sensor does increase greatly with stretching strain but decreases with extruding. The best fit of EPV for these currents, in proportion to s^1/2^, is linear in darkness but parabolic under illumination. This indicates that the strain current in darkness is mainly dominated by CR because the SBH depends linearly on the strain (discussed later).

In order to analyze these phenomena, let us first study the strain distribution within the SnS_2_/PDMS system, and calculate the electronic and optical properties of SnS_2_ under such strain. The stress and strain distributions are calculated by using a stationary solid-mechanics model performed in COMSOL Multiphysics tools (for details, see Supplementary Information). Supplementary Fig. 5a plots the simulation structure. The distribution of the von Mises stress (Supplementary Fig. 5b) for an applied stretching force shows that the SnS_2_ nanosheet encounters a larger stress than the PDMS due to the much higher Young’s modulus (56 gigapascals) of SnS_2_ compared to PDMS (0.75 megapascals), resulting in upwards bending displacement. However, the downwards bending displacement under an extruding force is not significant (Supplementary Fig. 5c). The principal-strain vector distribution on the SnS_2_ surface (Supplementary Fig. 5d) and the strain distributions (Supplementary Figs. 6 and 7) for the directions along and normal to the natural plane for a cross-sectional line, show that there is a shear strain normal to the natural plane. This stress-induced structural bending for a system with two contrasting hardness materials is schematically shown in Supplementary Fig. 6.

Based on these results, we calculated the band structure, dipole moment, absorption coefficient and refractive index for the unstressed structure and the normal- and shear-stressed structure using density functional theory (DFT) performed in the CASTEP code (details, see Supplementary Information). For a strain *s* < 1%, the bandgap variation (Supplementary Fig. 8 and Supplementary Table 1) is only tens of milli electron volts (meV), much smaller than the bandgap (2.2 eV) of SnS_2_, and therefore would not significantly change SnS_2_ resistance and its SBH. In addition, for a strain *s* < 1%, variations of absorption coefficient, and refractive index (Supplementary Fig. 9) are ~4.6% and ~0.2%, respectively, which would also not appreciably change the photoelectric effect in SnS_2_. Consequently, best candidate mechanism responsible for photo-induced giant gauge factor is the piezo-phototronic effect.

The dipole moment of a SnS_2_ nanosheet under normal and shear stresses was calculated using Mulliken charge, as shown in Supplementary Table 1, which indicates that there is a nonzero dipole moment distributed not only in the force direction but also in the *c* axis (*z*-direction) for the bending SnS_2_ nanosheet. The dipole (piezo) charge in the force direction appears on the sidewalls of the nanosheet, which would be ineffective at increasing the electric current in a top-gated configuration. While the *c*-axis piezo-charge is distributed over the top and bottom of the nanosheet creating a piezoelectric field $({{\rightharpoonup}\atop{{E_{\mathrm{pz}}}}})$ normal to the natural SnS_2_ plane. This field drives the carriers to randomly collide with the boundary of the nanosheet, based on the Drude model (Supplementary Fig. 10). We assume that the scattering plane at the boundaries for a weak van der Waals interaction is relatively smooth, therefore, the scattering can be treated as a simple mirror scatter. Thermally averaged, the trajectory of the random collisions under boundary scattering is semicircular when $|{{\rightharpoonup}\atop {{E_{\mathrm{a}}}}}| = | {{\rightharpoonup}\atop {{E_{\mathrm{pz}}}}}|$ but semi-elliptical when $| {{\rightharpoonup}\atop {{E_{\mathrm{a}}}}}| \ne | {{\rightharpoonup}\atop {{E_{\mathrm{pz}}}}} |$, where ${{\rightharpoonup}\atop {{E_{\mathrm{a}}}}}$ is the applied external electric field. In this situation, the mobility caused by the boundary scattering can be written as (for detailed numerical calculations, see the “Methods” section)

6 $$\mu _{{\mathrm{pz}}} = D/E_{\mathrm{z}}\tau _0,$$

where $E_{\mathrm{z}}$ is the electric field in z-direction and *τ*_0_ (~10 ps)^48^ is the phonon-limited relaxation time. However, according to the screening effect, the carriers in SnS_2_ do not wholly experience such an electric field but rather a screened one (*E*_sc_), which has the form (for detailed derivations, see “Methods”)

7 $$E_{{\mathrm{sc}}} = \frac{{DE_{\mathrm{z}}\kappa }}{{2{\mathrm{sinh}}\left( {\kappa D/2} \right)}}{\mathrm{cosh}}(\kappa z),$$

where $\kappa = 1/L_{\mathrm{D}}$, $L_{\mathrm{D}} = 1/\beta \sqrt {n_0}$ is the Debye length, and *β* (0.0212 m^1/2^ for the best fit) can be treated as a fitting parameter. There are two types of electric fields contributing to *E*_z_: (i) the piezoelectric field $E_{{\mathrm{pz}}} = \frac{{e_{{\mathrm{pz}}}s}}{{{\it{\epsilon }}_{\mathrm{s}}}}$ created by the piezocharge ($e_{{\mathrm{pz}}} = 0.005372\,{\mathrm{C}}/{\mathrm{m}}^2$) and (ii) the field $E_0 = \frac{{q_{{\mathrm{ext}}}}}{{{\it{\epsilon }}_{\mathrm{s}}}}$ by the external interface charge ($q_{{\mathrm{ext}}} = 0.078\,{\mathrm{C}}/{\mathrm{m}}^2$), leading to the boundary-scattering-limited mobility $\frac{1}{\mu } = \frac{{(E_0 + E_{{\mathrm{pz}}})\tau _0}}{D} = \frac{1}{{\mu _{\mathrm{z}}}} + \frac{1}{{\mu _{{\mathrm{pz}}}}}$, where

8 $$\mu _{{\mathrm{pz}}} = \frac{8}{{DE_{{\mathrm{pz}}}\kappa ^2\tau _0}}{\mathrm{sinh}}\left( {\kappa D_{{\mathrm{dp}}}/2} \right)\left[ {\tan {\,}^{ - 1}\left( {e{\,}^{\kappa D_{{\mathrm{dp}}}/2}} \right) - \tan {\,}^{ - 1}\left( {e{\,}^{ - \kappa D_{{\mathrm{dp}}}/2}} \right)} \right],$$

9 $$\mu _{\mathrm{z}} = \frac{8}{{DE_0\kappa ^2\tau _0}}\sinh \left( {\kappa D/2} \right)\left[ {\tan {\,}^{ - 1}\left( {e{\,}^{\kappa D/2}} \right) - \tan {\,}^{ - 1}\left( {e{\,}^{ - \kappa D/2}} \right)} \right].$$

Notably, (i) the piezo charge is separated by a distance of dipole length *D*_dp_ (0.295 nm), while the external charge is separated by the sheet thickness *D*. (ii) The reversed surface piezocharge under an extruding force offsets the external interface charge resulting in an increased mobility *μ* and current.

Based on Matthiessen’s rule, the total mobility can be calculated by the equation

10 $$\mu _t = \left( {1/\mu _0 + 1/\mu } \right)^{ - 1},$$

*μ*_0_ ($0.005\,{\mathrm{m}}^2/{\mathrm{V}} \cdot {\mathrm{s}}$)^49^ is the mobility in the absence of strain and external charge. The piezo-phototronic mobility arising via the screening effect is thickness and density dependent, which is consistent with that from interfacial scattering as observed in refs. ^50,51^. Under laser illumination the carrier density is increased by the extent of photo-generated carriers *n*_p_, which can be estimated by the relationship $n_{\mathrm{p}} = \eta P\tau /qE_{\mathrm{p}}D$, where the absorption efficiency $\eta = \frac{{4n_{{\mathrm{air}}}n_{{\mathrm{SnS}}_2}}}{{(n_{{\mathrm{SnS}}_2} + n_{{\mathrm{air}}})^2}}\left( {1 - e^{ - \alpha D}} \right)$ is approximated to 0.7461 due to a high measured absorption coefficient ($\alpha \sim 1.5 \times 10^7\,{\mathrm{cm}}^{ - 1}$)^52^ and the calculated refractive index $n_{{\mathrm{SnS}}_2} = 3.031\,{\mathrm{at}}\,{\mathrm{the}}\,{\mathrm{wavelength}}\,365\,{\mathrm{nm}},\,P\,{\mathrm{is}}\,{\mathrm{the}}\,{\mathrm{illuminating}}\,{\mathrm{power}}\,{\mathrm{density}}$, *τ*(74.1 ns)^53^ (The lifetime used herein is one order of magnitude higher than that measured in this reference, this difference maybe come from the different morphologies of flake SnS_2_ and nanoplate SnS_2_.) is the carrier lifetime, and *E*_P_ (3.4 eV for 365 nm light wavelength) is the photon energy. According to Eqs. (8)–(10), the calculated mobilities as a function of strain in darkness and under 365 nm laser illumination are indicated in Fig. 2c. Obviously, the mobility variation is much more sensitive to the strain under laser illumination than it is in darkness.

Apart from the modification of the carrier mobility, the carrier density injected by the photoelectric effect also modifies the equivalent doping density *N*_D_ in Eq. (2), that is $N_{\mathrm{D}}$ is replaced by *N*_D_ + *n*_p_. Moreover, the piezoelectric effect alters the build-in voltage in the depletion region of diode, and has the form ref. ^54^

11 $$\phi _{\mathrm{B}} = \phi _{{\mathrm{B}}0} + \frac{{qe_{{\mathrm{pz}}}W_{{\mathrm{pz}}}s}}{{2{\it{\epsilon }}_{\mathrm{s}}}},$$

where *ϕ*_B0_ (0.36 eV)^47^ is the barrier height under dark and unstressed conditions, and the sheet width *W*_p*z*_ (~0.295 nm for SnS_2_)^55^ of the piezoelectric charge is half of the *c* axis lattice constant.

**Fig. 3:**
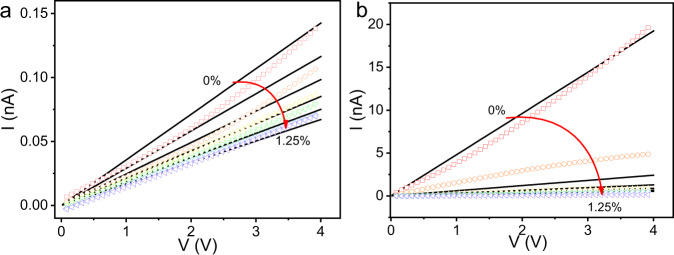
Model of measured sensor currents. Calculated currents (black lines) and measured currents (colored symbols) for a selection of strains for **a** dark and **b** 365 nm laser illuminating conditions.

The currents with respect to strain for dark and 365 nm laser illumination conditions are calculated by inserting Eqs. (3), (10), and (11) into Eq. (2) and then solving self-consistently. The calculated results are summarized in Fig. 3. Although the calculated results show some differences when compared with the experiment values, the theoretical model still demonstrates that the key mechanism in sensitivity enhancement is the screening effect of photocarrier on piezoelectric field, suppressing the boundary scattering. The overestimation of the strain effect (Fig. 3) in this simple classical model may be refined by considering the quantum interference effect, especially, in thinner region as shown in Supplementary Fig. 11a (the sensitivity as a function of SnS_2_ thickness). Supplementary Fig. 11a also shows that the thickness of the layered material dominates the degree of the screening effect and the sensitivity enhancement as observed in our measurements of the thinner WSe_2_ and MoSe_2_ samples (Supplementary Figs. 13 and 14), even with the different material parameters. It is worth noting that the observable nonzero current under the zero bias and zero strain in darkness may originate from the displacement current (*I*_disp_) in swept-measured mode^56^, according to equation $I_{{\mathrm{disp}}} = S\left( {{\it{\epsilon }}_{\mathrm{s}}\frac{{\partial E}}{{\partial t}} + \frac{{\partial e_{{\mathrm{pz}}}}}{{\partial t}}} \right)$^57^. The calculated sensitivities as a function of strain for dark and illuminated conditions are plotted in Supplementary Fig. 11b and c. One can see that the interplay of the piezoelectric and photoelectric (piezo-photonic) effects can result in a giant Gauge Factor of 3483. We also calculated the EPV due to strain, photoelectric, and piezo-photonic effects (Supplementary Fig. 12). One can also see that the piezo-photonic effect gives rise to the largest EPV, which further supports that the underlying behavior is due to the screening effect on piezoelectric field caused by photocarrier injection. It should be noted that nonequilibrium carriers can be injected by photo illumination, but also by other methods such as electrical gating, etc.^35^.

Our experiments further indicate the enhancement of the GF by photo illumination is a general phenomenon for Van der Waals semiconductor materials. Figure 2d summarizes the GF values of strain sensors based on a variety of Van der Waals semiconductor materials as a function of light power density, for materials including SnS_2_, monolayer WSe_2_, monolayer MoSe_2_, GaSe, and GeSe devices (Supplementary Figs. 13–16). The GF value of a GaSe based sensor is enhanced from 24 to 400. However, for monolayer WSe_2_ and monolayer MoSe_2_, the GF enhancement is less than 5. A possible explanation could be an increase of short-range interfacial scattering reducing the carrier mobilities in the monolayer devices. For GeSe, the *I–V* curves are quite linear for a variety of strain conditions, indicating a small Schottky barrier height between the electrodes and GeSe, as shown in Supplementary Fig. 15d. Note, the Gauge factors measured in dark are 64, 24, 15.2, and 1.7 for SnS_2_, GaSe, monolayer WSe_2_ and monolayer MoSe_2_, respectively. Those values are in the same range as those reported in the literature since the GF values reported for various types of van der Waals strain sensors are normally limited to well below 300^3,6,7,19,26–28,32,35^. We further summarized the change of Schottky barrier and GF for all of the above listed materials (Supplementary Fig. 17). It also suggests that the alteration of the SBH and CR by the piezo-photonic effect plays an important role in the enhancement of the GF value by photo illumination.

### Demonstration of real world applications of VdWLM strain sensors

**Fig. 4:**
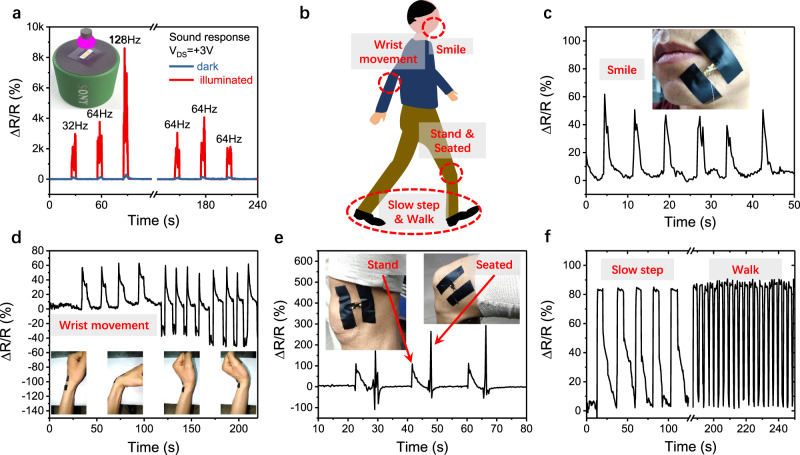
Demonstration of real world application of VdWLM strain sensor. **a** The performance of a SnS_2_ based strain sensor working under illumination when detecting vibrations induced by sound in comparison to signal when working in darkness. **b** Schematic of strain points produced by motion of a human body used for strain detection by SnS_2_ based devices. **c** The measured response produced by smile motion for sensor mounted on cheek, as shown in insert. **d** Response to wrist bending following positions shown below. **e** Sensor response to motion of standing and sitting corresponding to the insert image for sensor fixed on knee. **f** Signals from sensor on knee showing comparative response for slow and rapid walking.

To demonstrate the real world practical ability of our VdWLM based strain sensors, SnS_2_ based strain sensors were used to monitor tiny vibrations caused by sound and to capture a range of human motion, including facial, wrist, standing-sitting, and walking movements. Benefiting from giant GF values induced by illumination, the strain sensors are able to detect tiny vibrations. The inset of Fig. 4a shows a SnS_2_ based sensor on PDMS fixed on a rigid frame and the frame assembled above a sound box. Tiny vibrations were generated by playing short tones at different frequencies and were registered by the strain sensor. The sensor produces a strong and clear response under 365 nm light illumination due to the photon enhanced GF phenomenon, contrasting behavior is particularly evident when a comparison is made with the response of the same device under dark conditions, which show only a weak signal. For the response at 64 Hz, the change in relative resistance with illumination reaches ~1700%, a response ~21 times larger than that observed in darkness. To further demonstrate the application of VdWLMs based strain sensors for real world purposes, SnS_2_ based sensors on PET (polyethylene terephthalate) were also used to monitor different body movements as shown in Fig. 4b. In Fig. 4c, the movement of facial muscles is displayed for six repeated smiles, for a sensor attached to the cheek. For Fig. 4d the sensor monitors the signal generated by bending of a wrist through different positions. In Fig. 4e and f, the sensor is attached to a knee to monitor different ranges of motion. The movement of standing and sitting, is precisely tracked for three cycles, and slow and normal walking paces can be clearly distinguished by their periodic resistance behaviors.

**Fig. 5:**
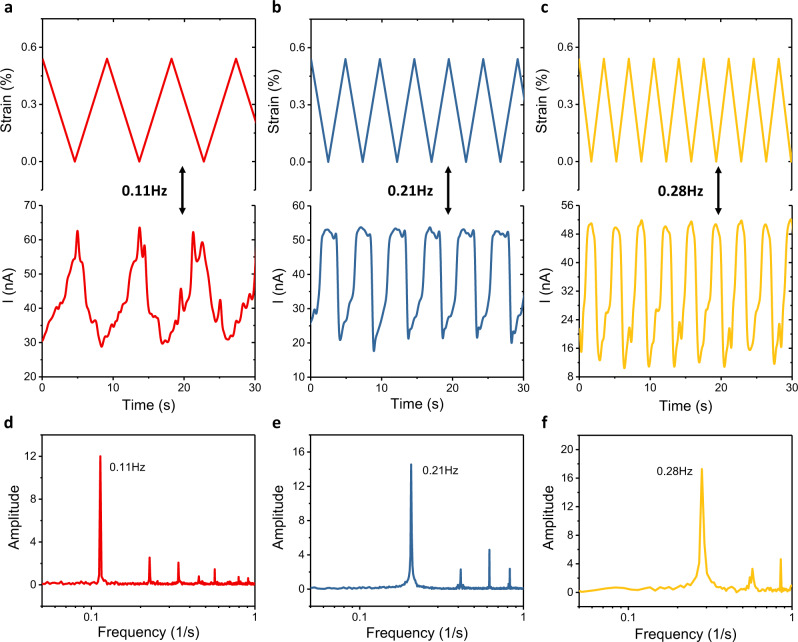
Dynamic strain sensing of SnS_2_ based strain sensors working under illumination. **a–c** The top row shows dynamic strain applied to a sensor at three different frequencies: **a** 0.11 Hz, **b** 0.21 Hz and **c** 0.28 Hz. The second row shows the current response corresponding to each dynamic strain state. **d**–**f** The bottom row shows frequencies calculated by Fourier transforms of *I–t* curve in figure (**a**–**c)**.

To demonstrate application potential of the strain sensors under repeated and varying strain conditions, we studied the dynamic response of the SnS_2_ based strain sensors under stretching strain. Similar to the static case, the sensor exhibits a higher strain response under illumination than in darkness. Figure 5a–c show the dynamic response of the SnS_2_ based device measured under illuminated conditions and an applied cyclic stretching strain (0–0.55% and of frequencies of 0.11, 0.21, and 0.28 Hz). This cyclic movement of strain is achieved by setting the strain-testing platform working at a certain cycling speed. In the second row of Fig. 5a–c displays the associated current trace for each case following their dynamic strain state. In Fig. 5d–f, corresponding Fourier transforms of each of *I* vs *t* plots reveal the frequency distribution of the registered strain data. The highest peak indicates their primary oscillation frequency, the other peaks represent higher order natural harmonics and noise within the system.

## Discussion

We have presented an interesting and apparently universal strategy to tune and enhance the GF of strain sensors based on Van der Waals materials. The GF values can be tuned over a very large range, over 2 orders of magnitude, and a gauge factor value as high as 3933 is achieved for a SnS_2_ based strain sensor, which has the potential to be further enhanced by increasing light power density. Two types of real world applications, i.e., detecting tiny vibrations caused by sound and daily movements of the human body, were also demonstrated.

## Methods

### Device fabrication

The SnS_2_ nanoflakes were transferred by polydimethylsiloxane (PDMS) stamps from a fully covered Si substrate, produced by chemical vapor deposition (CVD)^58^, to a PDMS bar (Supplementary Fig. 18c). The GaSe and GeSe nanoflakes were mechanically exfoliated from bulk crystals, provide by HQ graphene, and then transferred to a PDMS bar. The monolayer WSe_2_ was easily removed from Si substrate by immersion in deionized water and floated onto PDMS (Supplementary Fig. 18a)^59^. The monolayer MoSe_2_ was transferred by wet transfer^60^, including: spin coating a polymethyl methacrylate (PMMA) layer onto the SiO_2_/Si growth substrate for MoSe_2_, separation of the PMMA/MoSe_2_ and substrate by a KOH etchant solution, floating MoSe_2_ crystal onto a PDMS bar and removing the PMMA layer with acetone (Supplementary Fig. 18b). A 30 μm diameter aluminum wire was placed above the target crystal as a shadow mask^58^. The ends of the wire were fixed with adhesive tape or silver paint. Under a <9 × 10^−6^ Pa vacuum condition, 10 nm thick Ti and 140 nm thick Au film were deposited by thermal evaporation. Finally, the adhesive tape and wire were removed.

### Numerical calculations

The piezoelectric field combined with the laterally applied field forces the conduction electrons and holes to move in a semicircular or semi-elliptical trajectory due to boundary scattering (Supplementary Fig. 10). Solving the equations of motion in consideration of defect- and phonon-limited relaxations yields an average velocity $\bar v = qE_{{\mathrm{pz}}}\tau _0/m^ \ast$, where m* is the effective electron mass of SnS_2_. Since the relaxation time is the average time interval between two successive scattering events, the relaxation time caused by the strain is $\tau _{{\mathrm{pz}}} = m^ \ast D/qE_{{\mathrm{pz}}}\tau _0$, and thus yields the mobility

12 $$\mu _{{\mathrm{pz}}} = q\tau _{{\mathrm{pz}}}/m^ \ast = D/E_{{\mathrm{pz}}}\tau _0.$$

The carrier density in VdWLM as a function of local potential is

13 $$n\left( z \right) = N_{\mathrm{c}}e^{ - \left[ {E_{\mathrm{c}} - E_{{\mathrm{f}}0} + q\varphi (z)} \right]/k_{\mathrm{B}}T},$$

where *N*_c_ is the effective conduction-band density of states, *E*_c_ is the conduction band edge, *E*_f0_ is the Fermi energy in the absence of a local potential *φ*(*x*). As a first-order approximation, Eq. (13) can be written as

14 $$n\left( z \right) \cong n_0 - \frac{{n_0}}{{V_{\mathrm{T}}}}\varphi (z);$$

$V_{\mathrm{T}} = k_{\mathrm{B}}T/q$ is the thermal potential. Then, the induced charge distribution is

15 $$\Delta n = n\left( z \right) - n_0 = - n_0\varphi /V_{\mathrm{T}}.$$

Thus, we get the Poisson equation $\frac{{d^2\varphi }}{{dz^2}} = \frac{{qn_0}}{{{\it{\epsilon }}_sV_{\mathrm{T}}}}\varphi$. According to the boundary conditions $n\left( 0 \right) = 0$ (or $\left. {\frac{{d^2\varphi }}{{dz^2}}} \right|_{z = 0} = 0$) and $\varphi \left( {D/2} \right) = - V_0/2$ for a center-symmetric structure, the solution becomes

16 $$\varphi \left( z \right) = \frac{{ - V_0}}{{2{\mathrm{sinh}}(\kappa D/2)}}{\mathrm{sinh}}(\kappa z),$$

for $- D/2 \le z \le D/2$, where $\kappa = 1/L_{\mathrm{D}}$, $L_{\mathrm{D}} = \sqrt {\frac{{{\it{\epsilon }}_{\mathrm{s}}V_{\mathrm{T}}}}{{qn_0}}} = \frac{1}{{\beta \sqrt {n_0} }}$ is the Debye length, and $V_0 = DE_{\mathrm{z}}$ is the unscreened potential due to *E*_z_. The screened electric field in the material is

17 $$E_{{\mathrm{pz}}}\left( z \right) = \frac{{V_0\kappa }}{{2{\mathrm{sinh}}(\kappa D/2)}}{\mathrm{cosh}}(\kappa z).$$

According to the relationship $\frac{{dz}}{{dt}} = qE_{{\mathrm{pz}}}(z)\tau _0/m^ \ast$, the relaxation time can be calculated by

18 $$\tau _{{\mathrm{pz}}} = \frac{{2m^ \ast {\mathrm{sinh}}(\kappa D/2)}}{{q\tau _0DE_{\mathrm{z}}\kappa }}\int_{ - D/2}^{D/2} \frac{1}{{{\mathrm{cosh}}(\kappa z)}}dz.$$

Solving Eq. (18) and using its result to calculate the mobility, one yields Eqs. (8) and (9). The calculated screened potential and screened electric field in the nanosheet are plotted in Supplementary Fig. 19.

## Supplementary information

Supplementary Information

## Data Availability

The data that support the findings of this study are available from the corresponding author upon request.
