# Supplementary material for: Giant gauge factor of Van der Waals material based strain sensors
Source: Nat Commun. 2021 Apr 1;12:2018. doi: 10.1038/s41467-021-22316-8 (PMC8016834; doi:10.1038/s41467-021-22316-8)
Supplement: Supplementary file 1 — Supplementary Information [file 41467_2021_22316_MOESM1_ESM.pdf]

Supplementary information

Giant Gauge factor of Van der Waals material based strain sensors

Wenjie Yan[1], Huei-Ru Fuh[2,3], Yanhui Lv[1], Ke-Qiu Chen[4], Tsung-Yin Tsai[5], Yuh-Renn Wu[5], Tung-Ho Shieh[6], Kuan-Ming Hung[7,], Juncheng Li[1], Duan Zhang[8], Cormac Ó Coileáin[9], Sunil K. Arora[10], Zhi Wang[1], Zhaotan Jiang[1], Ching-Ray Chang[2], and Han-Chun Wu[1,*]*

[1]School of Physics, Beijing Institute of Technology, Beijing 100081, P. R. China

[2]Department of Physics, National Taiwan University, Taipei 106, Taiwan

[3]Department of Chemical Engineering & Materials Science, Yuan Ze University, Taoyuan City 320, Taiwan

[4]Department of Applied Physics, School of Physics and Electronics, Hunan University, Changsha 410082, P. R. China

[5]Graduate Institute of Photonics and Optoelectronics and Department of Electrical Engineering, National Taiwan University. Taipei, 10617, Taiwan

[6]Department of Intelligent Robotics Engineering, Kun-Shan University, Tainan 710, Taiwan

[7]Department of Electronics Engineering, National Kaohsiung University of Science and Technology, Kaohsiung 807, Taiwan

[8]Elementary Educational College, Beijing key Laboratory for Nano-Photonics and Nano-Structure, Capital Normal University, Beijing 100048, P. R. China

[9]Centre for Research on Adaptive Nanostructures and Nanodevices (CRANN) and Advanced Materials and Bioengineering Research (AMBER), School Chemistry, Trinity College Dublin, Ireland

[10]Centre for Nanoscience and Nanotechnology, Panjab University, Chandigarh-160014, India

* Address correspondence to: wuhc@bit.edu.cn; kmhung@nkust.edu.tw

Supplementary Note 1: Calculations of stress and strain distributions

The stress and strain distributions of the structure of a thin SnS_2 layer ($30\mu m \times 15\mu m \times 0.2\mu m$) on PDMS ($100\mu m \times 50\mu m \times 5\mu m$) as shown in Figure S5a were calculated by applying a stationary solid-mechanics model performed in COMSOL Multiphysics software tool. The parameters of mass density, Young's modulus and Poisson ratio for PDMS (for SnS_2) are $970\ kg/m^3$ $(3690\ kg/m^3)$, $0.75\ MPa$ (56 GPa)[1] and 0.49 (0.25), respectively. The Young's modulus and Poisson ratio for PDMS are standard parameters in COMSOL tools. The boundary surface at $y = 0$ is fixed and that at $y = 100\ \mu m$ applies a pressure of 0.5 MPa (-0.5 MPa) for stretching (extruding) the PDMS. Other surfaces are free of constrains. The calculated results of the structure under stretching and extruding forces on the PDMS exhibit the same stress distributions in the SnS_2 nanosheet (Figures S5b and S5c). The nanosheet encounters a much higher stress than the PDMS, due to the higher Young's modulus of SnS_2 (56 GPa) relative to PDMS (0.75 MPa), and experiences an in-plane strain along the direction of applied force and a shear strain normal to the top natural plane as shown in Figures S5d, S6b and S6c. As shown in Figures S6, the bottom surface of nanosheet bounded to PDMS encounters a direct stress from PDMS in force direction, however, the top surface is free of constraints, resulting in the former experiencing a larger strain than latter.

Supplementary Note 1: Calculations of the electronic and optical properties of SnS_2 under various normal

Figure S7b shows that the shear strain is non-uniformly distributed along the direction normal to the plane. This means that the SnS_2 nanosheet not only encounters a tensile strain in the force-direction under a stretching force but also concurrently bends upward (Figures S7g and S7e). Conversely, the SnS_2 nanosheet encounters a compressive strain and bends downward under an extruding force (Figures S7a, S7c, S7d and S7f).

The calculations of the electronic and optical properties of SnS_2 under various normal stresses (along the y-axis of the crystal) and shear stresses (along the c-axis) using density functional theory with a plane wave basis set (the cut-off energy is 650eV) and geometry optimization was performed in the CASTEP code. The exchange and correlation energies used the revised Perdew-Burke-Ernzerhof (RPBE) functional with the generalized gradient approximation (GGA), and the norm-conserving method for pseudopotential estimation was used. The applied stress is achieved by setting the stress-tensor components of normal stress σ_y and shear stress σ_{yz}, the reference coordinates are shown in Figure S5d, and hard compressibility was selected during geometric optimization. The calculated results are plotted in Figure S8. The vector of the dipole moment ($\vec{P}$) was calculated by the product of the Mulliken charge and the distance between the S and Sn atoms. The variation of the dipole moment can be estimated by $\Delta\vec{P} = \vec{P}(s) - \vec{P}(0)$. The calculated results are listed in Supplementary Table 1.

Supplementary Tables

	No stress	$\sigma_y = 0.5\ GPa$<br>$\sigma_{yz} = 0.1\ GPa$	$\sigma_y = 1\ GPa$<br>$\sigma_{yz} = 0.2\ GPa$
Energy gap (eV)	2.18	2.172	2.159
$\Delta\vec{P}\ (10^{-3} q \cdot nm)$	$\vec{0}$	$-0.65\hat{x} \mp 1.05\hat{y} + 0.5\hat{z}$	$-0.7\hat{x} + 1.54\hat{y} + 0.89\hat{z}$
Strain (%)	0	0.45	0.97
Refractive index n	3.03	3.02	3.025
Absorption coefficient $(10^4 cm^{-1})$	1.506	1.468	1.437

Supplementary Table 1. Calculated Energy gap, $\Delta\vec{P}$, Refractive index n and Absorption coefficient under various strain conditions.

Supplementary Figures

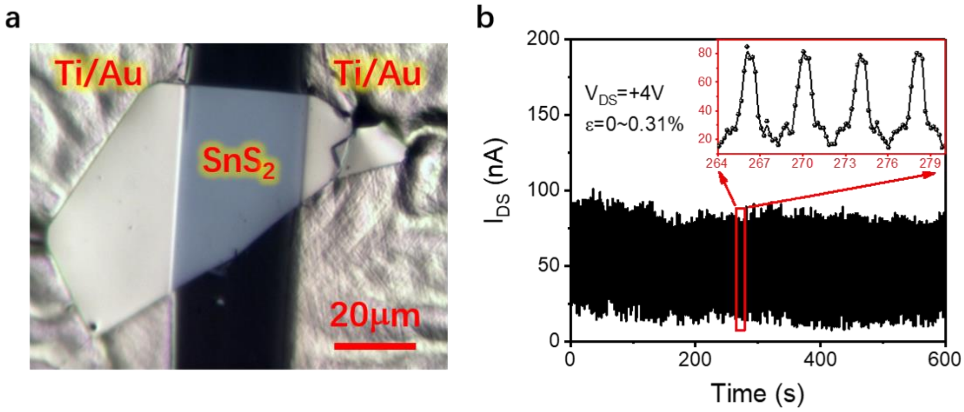

Supplementary Figure 1. Supplemental data for SnS_2 based strain sensor. a, Optical image of a SnS_2-based strain sensor. **b**, Current response of SnS_2 based strain sensor measured under 365 nm laser illumination at a bias voltage of 4V over many cycles of repeatedly stretching the substrate. The inset image is an enlargement of the cyclic test around ~273s.

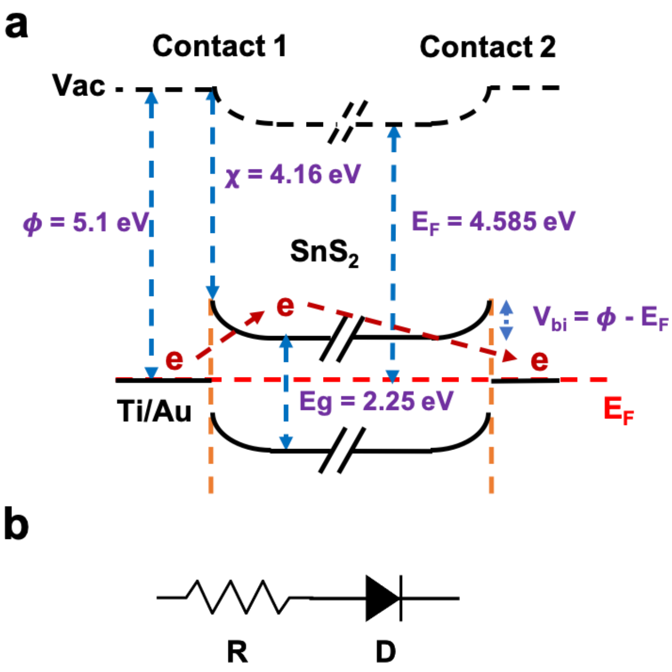

Supplementary Figure 2. (a) Schematic plots of the band profile for SnS_2 with Ti/Au contacts, and (b) the equivalent circuit.

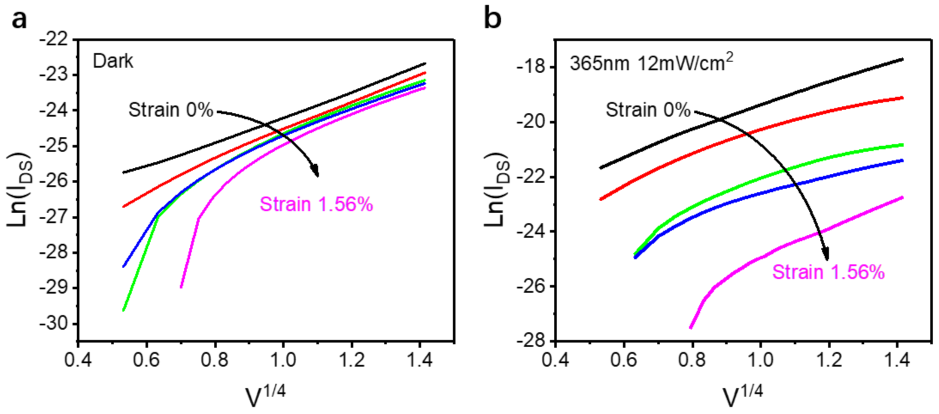

Supplementary Figure 3. LnI_{DS} plotted as a function of $V^{1/4}$. **a**, Logarithm of current (I_{DS}) versus $V^{1/4}$ curves calculated from the data in Fig. 2b. **b**, Logarithm of I_{DS} versus $V^{1/4}$ curves calculated from the data in Fig. 2c.

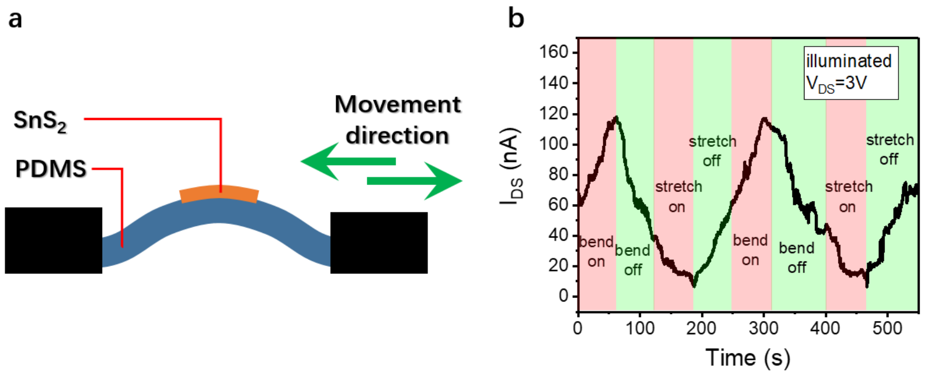

Supplementary Figure 4. Continuous bending and stretching test for a SnS_2 based strain sensor. **a**, A schematic of SnS_2 based strain sensor in a continuous strain test. The SnS_2 nanosheet is bent when the whole device compresses, and it is stretched as the whole device is stretched. **b**, Current responses of SnS_2 device in continuous strain test following the mechanical movement as illustrated in Fig. S4a.

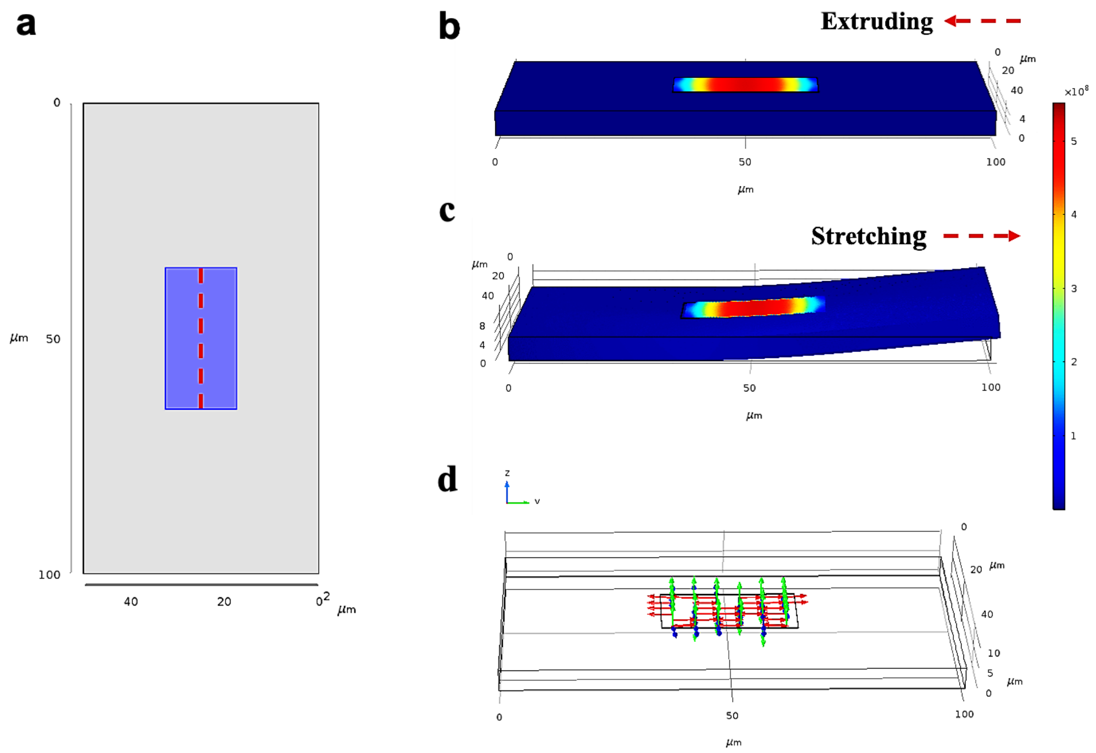

Supplementary Figure 5. (a) Structure schematic. The red-dashed line indicates the position of the cross-sectional strain profile in Figure S6. The distribution of von Mises stress in units of N/m^2 for (b) extruding force and (c) stretching force. (d) The principal strain vectors at the top of SnS_2 surface include the strain vectors along the direction normal (green) to the natural plane and those (red and blue) within the plane.

a

b

c

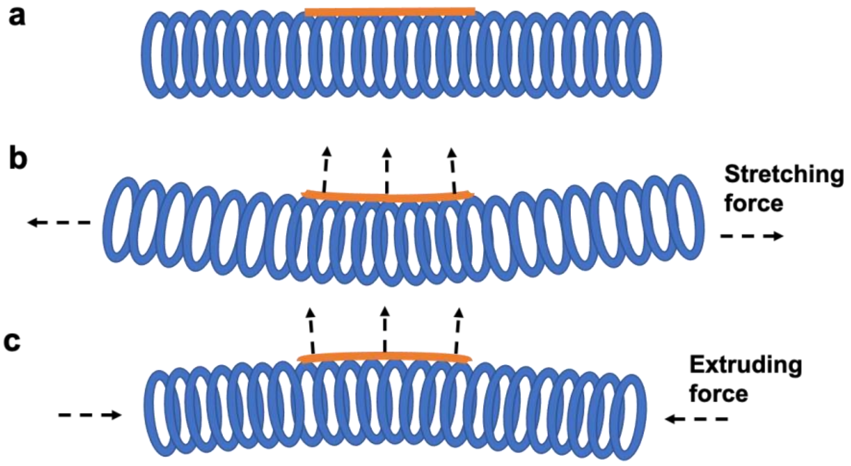

Supplementary Figure 6. Illustrative schematic of composite structure bending of (a) no stress, (b) stretching force and (c) extruding force, where the blue circle represents the PDMS and the broad-khaki-color line represents the SnS_2, and the black arrows indicate the directions normal to the plane.

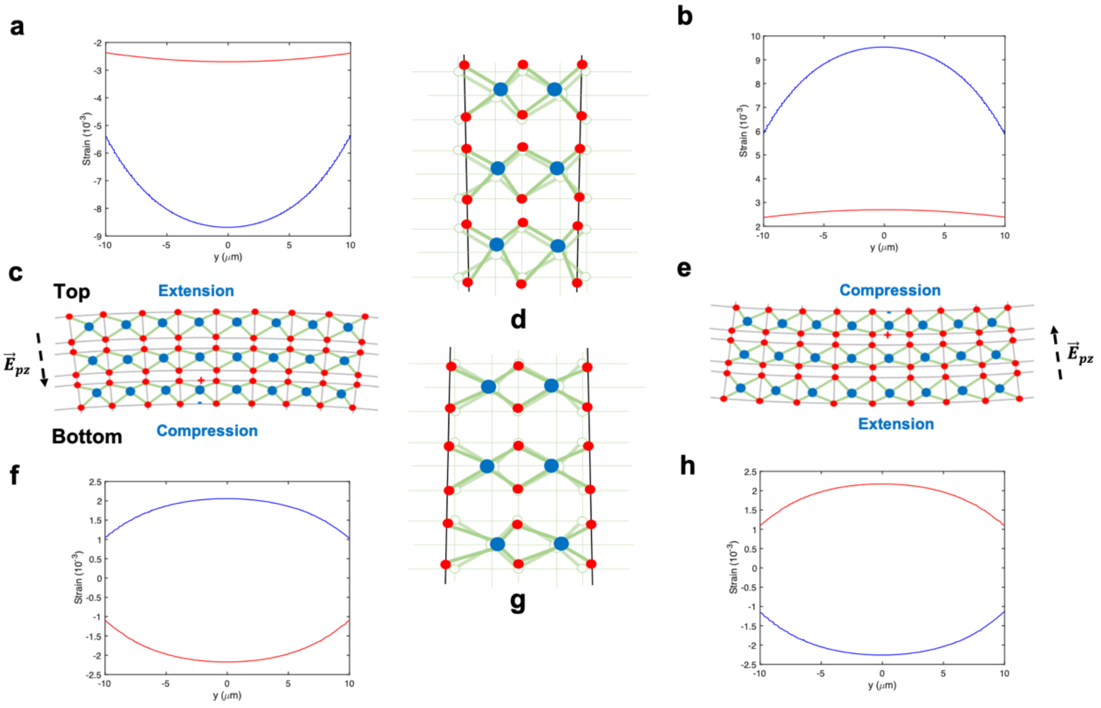

Supplementary Figure 7. (a) and (b) indicate the strain distributions in force direction (the red vectors in Figure S5d), along the cross-section line as shown in Figure S5a, for extruding and stretching forces, respectively, where the blue (red) line indicates the strain at the bottom (top) of SnS_2 nanosheet. Bending structures of SnS_2 nanosheet for (c) extruding and (e) stretching forces on PDMS. For clarity, enlarged plots of bending structure for (d) extruding and (g) stretching forces are shown, where the unshaded circles indicate the locations of unstressed atoms. (f) and (h) the strain distribution in the direction normal to the top of the natural plane for extruding and stretching forces, respectively. Wherein the blue line shows the strain at the bottom of the SnS_2 layer and the red line the strain at the top of the SnS_2 layer.

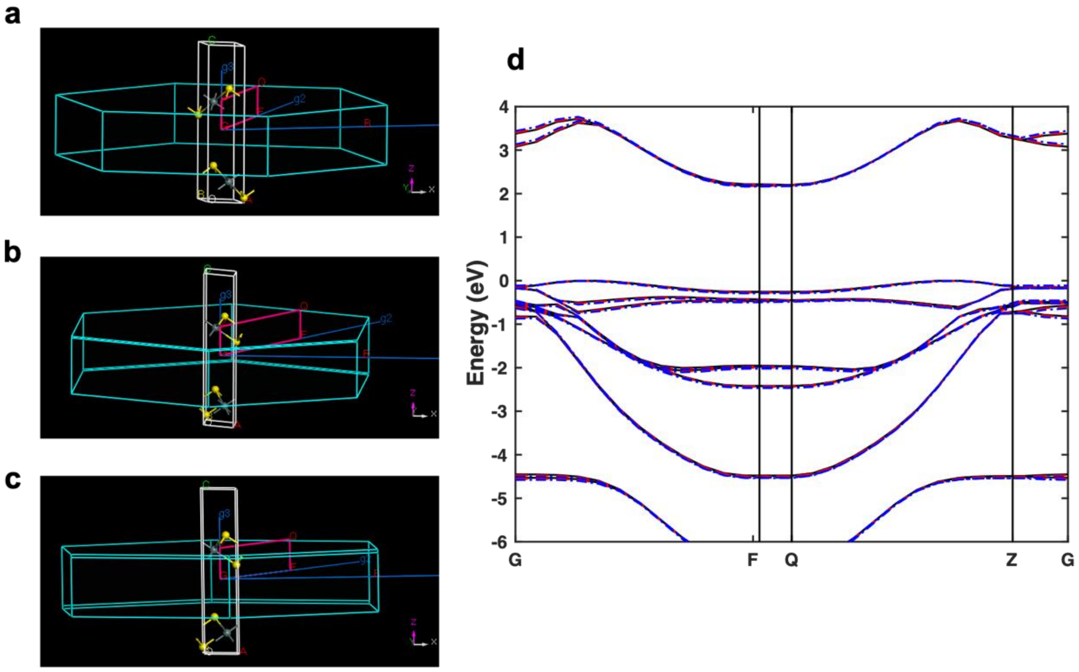

Supplementary Figure 8. The plots of (a) unstressed crystal structure, (b) stressed structure (with the stress components of $\sigma_y = 0.5\, GPa$ and $\sigma_{yz} = 0.1\, GPa$) and (c) stressed structure (with $\sigma_y = 1.\, GPa$ and $\sigma_{yz} = 0.2\, GPa$), and their reciprocal lattice (green lines), where grey-solid circle is the tin atom and yellow-solid circle is the sulfur atom. (d) Plots of the band structures (black lines: no stress; red-dashed lines: stress with $\sigma_y = 1.\, GPa$ and $\sigma_{yz} = 0.2\, GPa$; and blue-dash-dotted line: stress with $\sigma_y = 1.\, GPa$ and $\sigma_{yz} = 0.2\, GPa$).

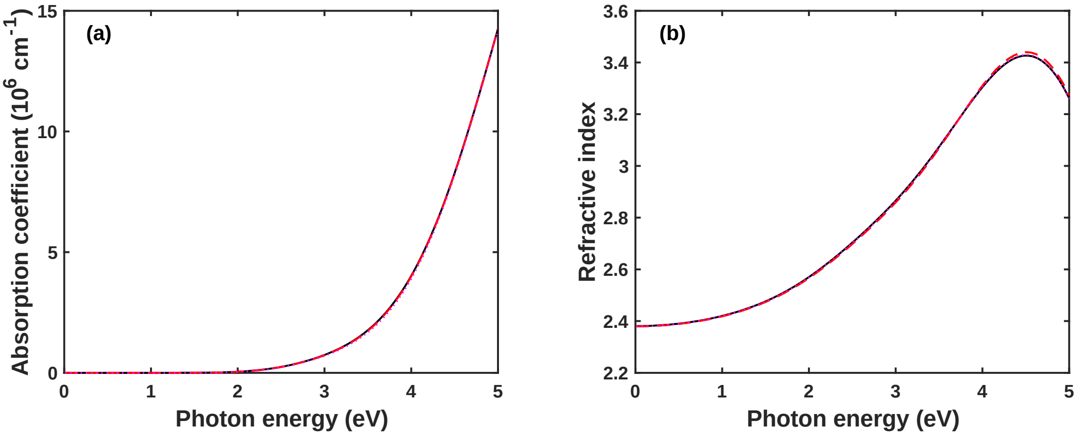

Supplementary Figure 9. (a) The calculated absorption coefficients for no stress (black) and stresses of $\sigma_y = 0.5\ GPa$ (red-dotted line) and $\sigma_y = 1\ GPa$ (red line). (b) The calculated refractive index for no stress (black) and stresses of $\sigma_y = 0.5\ GPa$ (red-dotted line) and $\sigma_y = 1\ GPa$ (red-solid line).

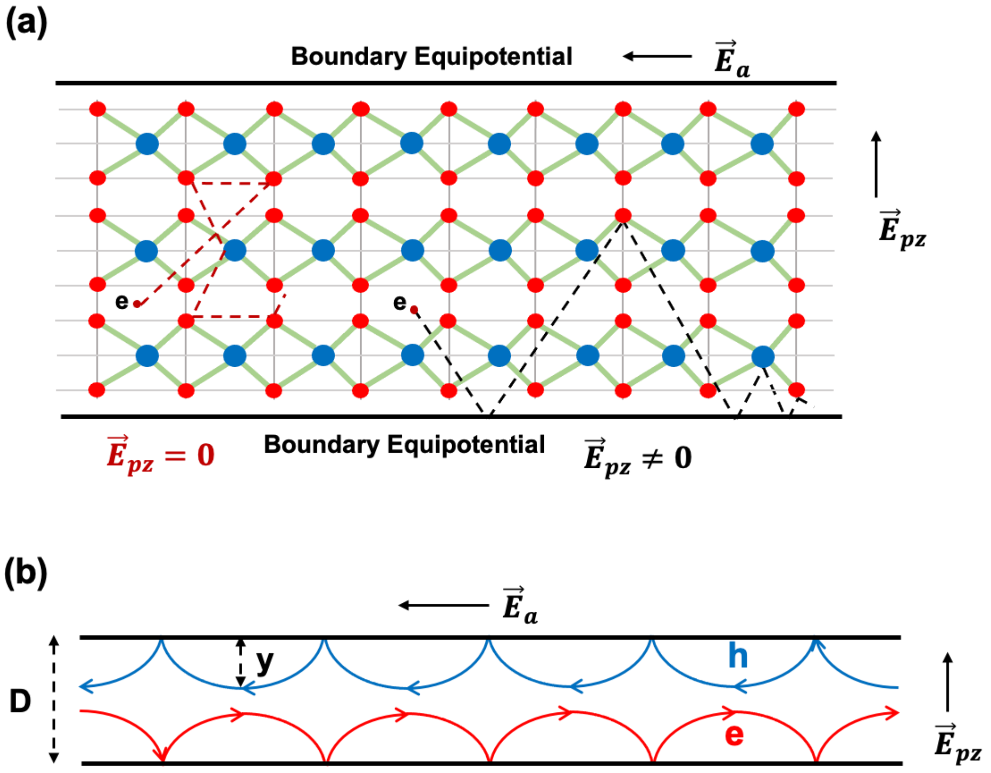

Supplementary Figure 10. (a) Schematic of the path for scattered electron transport for $\vec{E}_{pz} = 0$ (red line) and $\vec{E}_{pz} \neq 0$ (black line). (b) The thermally averaged path for scattered electrons (red line) and holes (blue line).

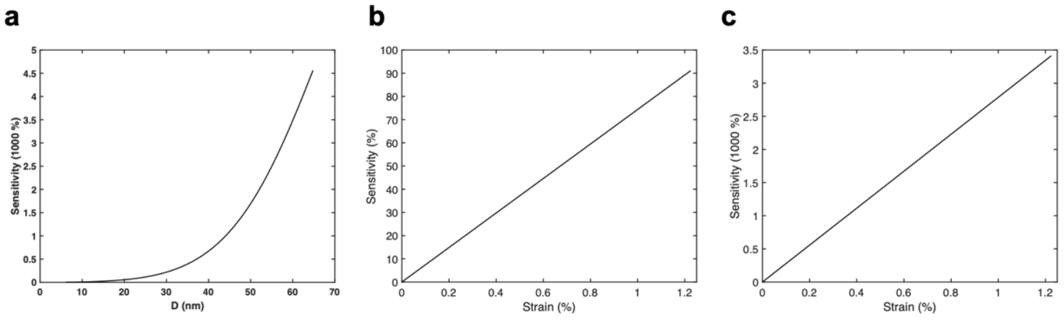

Supplementary Figure 11. (a) plot of the calculated sensitivity as a function of SnS_2 thickness at $V = 4\,V$ and $s = 1.25\%$. (b) and (c) plot of the calculated sensitivities as a function of strain in darkness and under 365 nm light illumination, respectively.

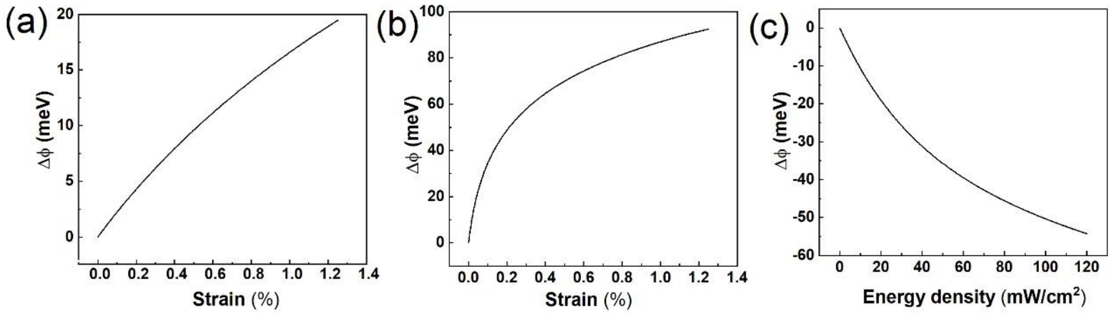

Supplementary Figure 12. (a) Calculated $\Delta\phi$ as a function of strain in darkness and (b) under 365 nm light illumination. (c) Calculated $\Delta\phi$ as a function of incident-light power density P under a strain of 1.25 %.

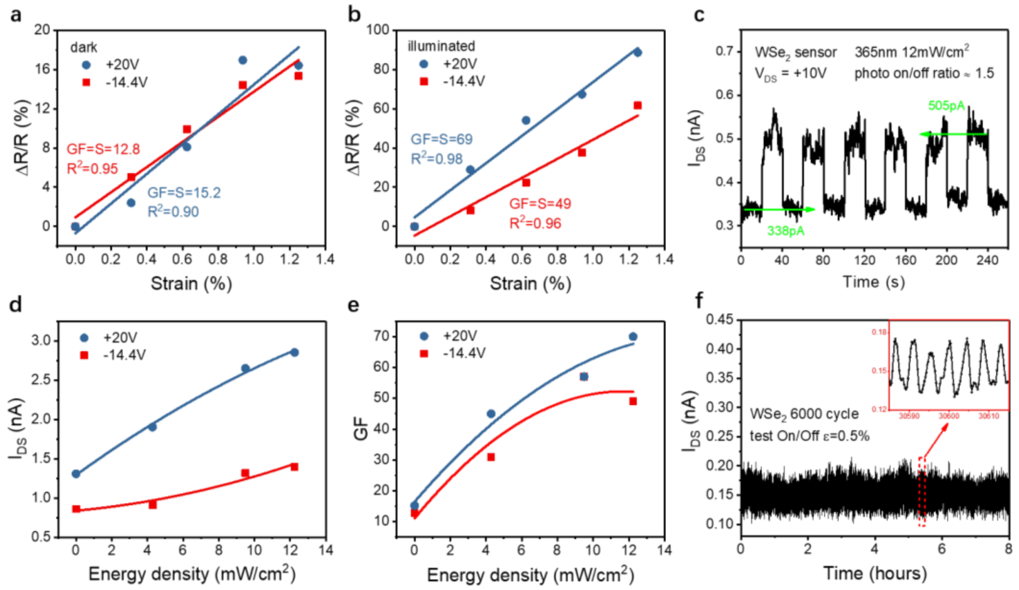

Supplementary Figure 13. A series of tests on WSe_2 based strain sensor. a, The function of $\Delta R/R_0$ versus strain of a sensor measured in darkness. Linear fitted blue and red lines show the GF is 15.2 at +20V and 12.8 at -14.4V. **b**, The function of $\Delta R/R_0$ versus strain of the sensor measured under 365nm illumination. Linear fitted blue and red lines show the GF is 69 at +20V and 49 at -14.4V. **c**, Light on/off tests for WSe_2 device at +10V. **d**, Current responses for light intensity on this device. **e**, GF evolution as light intensity increases for this device. **f**, 6000 cycle strain on/off test for WSe_2 device.

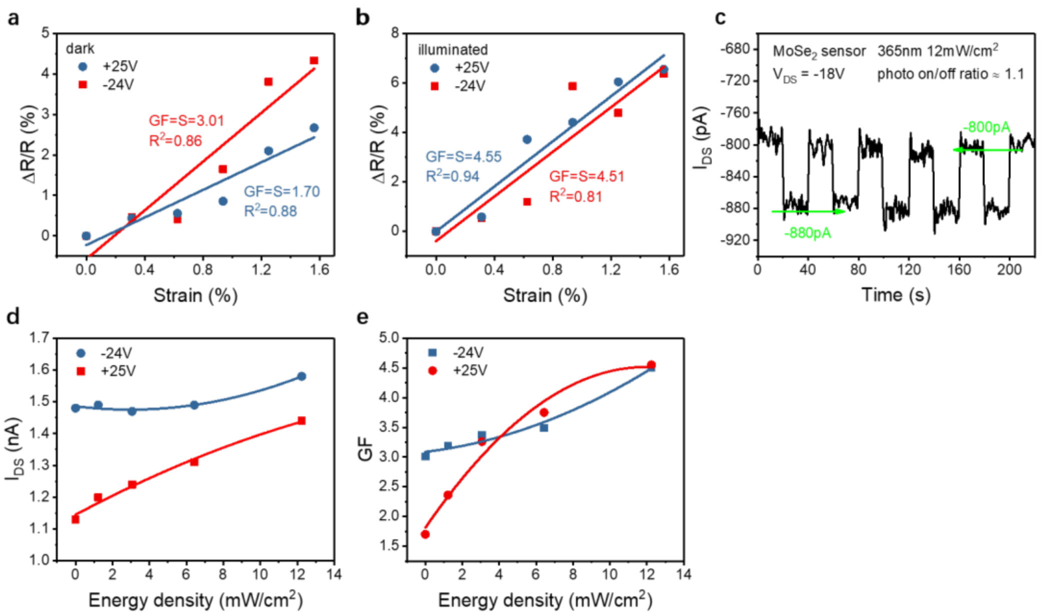

Supplementary Figure 14. A series of tests on $MoSe_2$ based strain sensor. a, The function of $\Delta R/R_0$ versus strain of a sensor measured in darkness. Linear fitted blue and red lines show the GF is 1.70 at +25V and 3.01 at -24V. **b**, The function of $\Delta R/R_0$ versus strain of the sensor measured under 365nm illumination. Linear fitted blue and red lines show the GF is 4.55 at +25V and 4.51 at -24V. **c**, Light on/off tests of this device. **d**, Current responses with light intensity for this device. **e**, GF evolution as light intensity increases for $MoSe_2$ device.

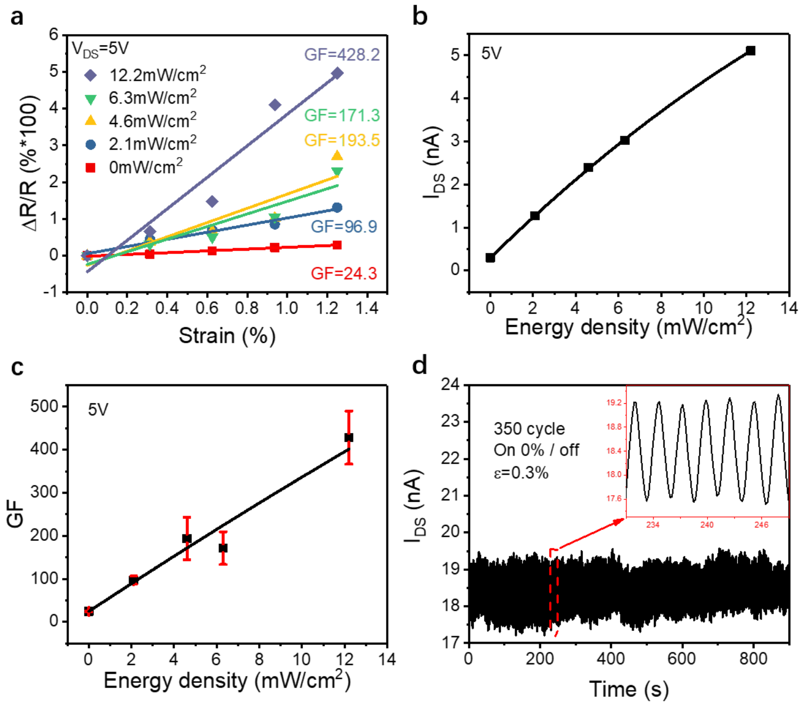

Supplementary Figure 15. A series of tests on GaSe based strain sensor. a, The development of relative resistance with different illumination conditions from 0 to 12mW/cm² at +5V. **b**, Current responses with light intensity for this device. **c**, GF evolution as light intensity increases for this device. **d**, 350 cycle strain on/off test for GaSe.

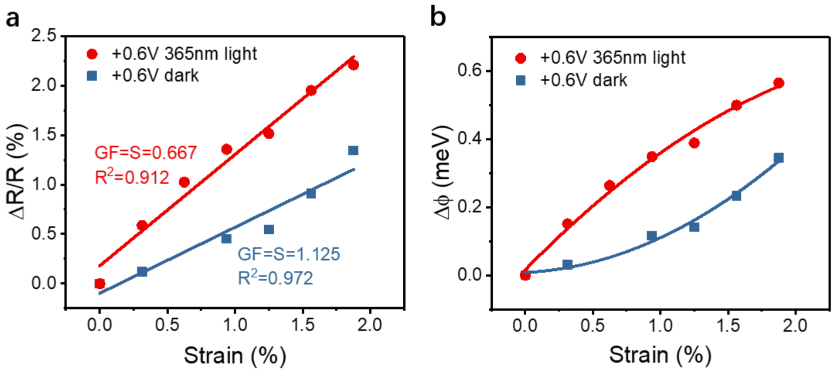

Supplementary Figure 16. A series of tests for GeSe based strain sensor. a, The development of relative resistance with different illumination conditions at +0.6V. **b**, Calculated variation of barrier height ($\Delta\phi$) with strain (s).

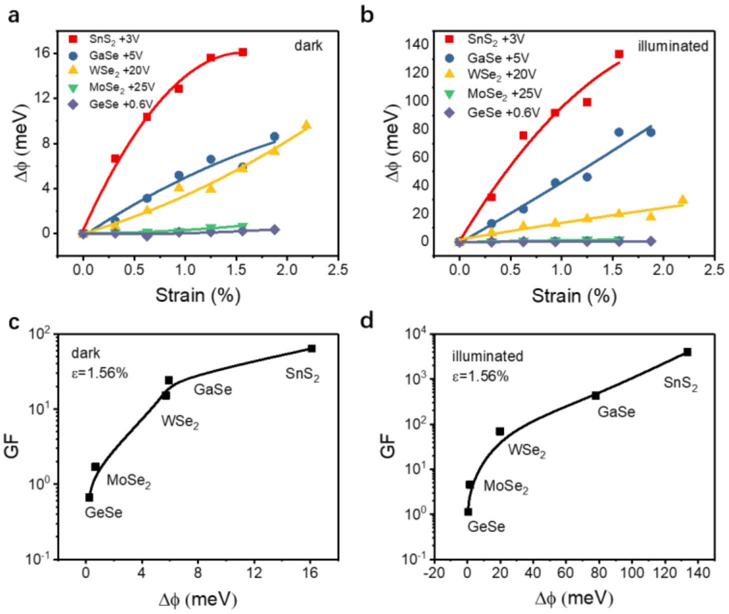

Supplementary Figure 17. Variation of Schottky barrier height under different strain state for several types of Van der Waals semiconducting materials including SnS_2, GaSe, GeSe, monolayer WSe_2 and monolayer $MoSe_2$. a-b, Calculated changes of barrier height of variety types of Van der Waals materials based strain sensors with strain (ε) applied with and without external illumination. **c-d**, Demonstration of the relationship between GF and variation of Schottky barrier height, induced by applying 1.56% strain, with and without external illumination.

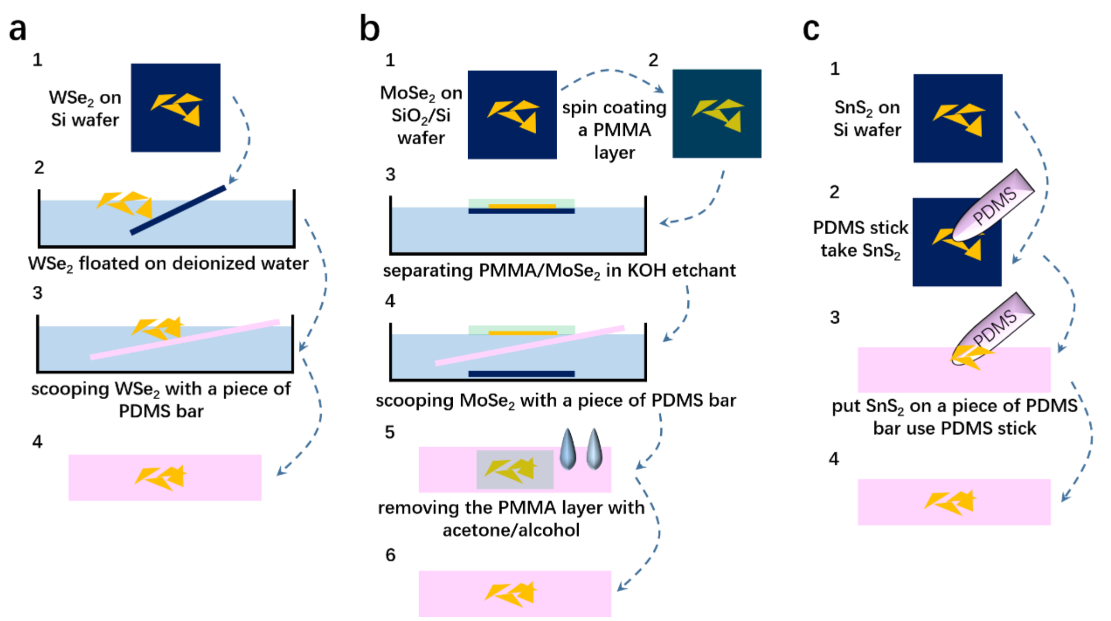

Supplementary Figure 18. Schematic of Van der Waals materials transfer procedures.

a. Wet transfer process for WSe_2: 1 Preparing monolayer WSe_2 crystal on Si wafer by CVD. 2 WSe_2 crystal separates from Si wafer and floats on the surface of deionized water. 3 Scooping WSe_2 with a piece of PDMS. 4 WSe_2 crystal transferred onto the PDMS. **b.** Wet transfer process for $MoSe_2$: 1 Preparing monolayer $MoSe_2$ crystal on SiO_2/Si wafer by CVD. 2 Spin coating a PMMA layer on wafer. 3 KOH solution serves as SiO_2 etchant to separate PMMA/$MoSe_2$ and Si. 4 Scooping $MoSe_2$ with a piece of PDMS. 5 The PMMA layer is removed by immersion in acetone for 6 hours and, then immersing in alcohol for 1 hour. 6 $MoSe_2$ crystal transferred onto the PDMS bar. **c.** Dry transfer for SnS_2: 1 Preparing SnS_2 crystal on Si wafer by CVD. 2 Picking up several SnS_2 nanosheets with PDMS stick. 3 Rubbing on a piece of PDMS bar with the PDMS stick to place down a SnS_2 crystal. 4 SnS_2 crystal transferred onto PDMS.

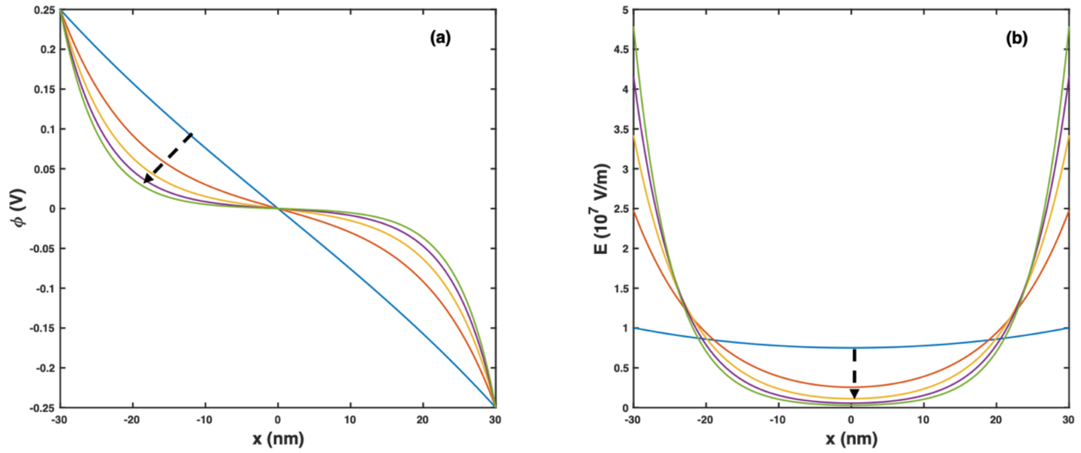

Supplementary Figure 19. (a) Plots of the screened potentials and (b) screened electric field, for $\kappa = 2.6618 \times 10^7 \sim 1.9157 \times 10^8\ m^{-1}$ (with equally incremental density of $\Delta n = 2 \times 10^{19}\ m^{-3}$) under $V_0 = 0.5$ and $T = 300\ K$.
